# Robotic *versus* open resection for colorectal liver metastases in a “referral centre Hub&Spoke learning program”. A multicenter propensity score matching analysis of perioperative outcomes

**DOI:** 10.1016/j.heliyon.2024.e24800

**Published:** 2024-01-17

**Authors:** Aldo Rocca, Pasquale Avella, Andrea Scacchi, Maria Chiara Brunese, Micaela Cappuccio, Michele De Rosa, Alberto Bartoli, Germano Guerra, Fulvio Calise, Graziano Ceccarelli

**Affiliations:** a Department of Medicine and Health Science “V. Tiberio”, University of Molise, Campobasso, Italy; b Hepatobiliary and Pancreatic Surgery Unit, Pineta Grande Hospital, Castel Volturno, Caserta, Italy; c Department of Clinical Medicine and Surgery, University of Naples “Federico II”, Naples, Italy; d General Surgery Department, University of Milano-Bicocca, Milan, Italy; e General Surgery Department, ASL 2 Umbria, San Giovanni Battista Hospital, Foligno, Italy

**Keywords:** Colorectal liver metastases, Open liver surgery, Robotic liver resection, Hub and spoke program, Learning program

## Abstract

**Background:**

Surgical resection is still considered the optimal treatment for colorectal liver metastasis (CRLM). Although laparoscopic and robotic surgery demonstrated their reliability especially in referral centers, the comparison between perioperative outcomes of robotic liver resection (RLR) and open (OLR) liver resection are still debated when performed in referral centers for robotic surgery, not dedicated to HPB. Our study aimed to verify the efficacy and safety of perioperative outcomes after RLR and OLR for CRLM in an HUB&Spoke learning program (H&S) between a high volume center for liver surgery and high volume center for robotic surgery.

**Methods:**

We analyzed prospective databases of Pineta Grande Hospital (Castel Volturno) and Robotic Surgical Units (Foligno-Spoleto and Arezzo) from 2011 to 2021. A 1:1 propensity score matching (PSM) was performed according to baseline characteristics of patients, solitary/multiple CRLM, anterolateral/posterosuperior location.

**Results:**

383 patients accepted to be part of the study (268 ORL and 115 RLR). After PSM, 45 patients from each group were included. Conversion rate was 8.89 %. RLR group had a significantly lower blood loss (226 *vs.* 321 ml; *p=0.0001*), and fewer major complications (13.33 % *vs.* 17.78 %; *p=0.7722*). R0 resection was obtained in 100% of OLR (*vs.95.55%, p =0.4944*. Hospital stay was 8.8 days in RLR (*vs.* 15; *p=0.0001*).**Conclusion:** H&S represents a safe and effective program to train general surgeons also in Hepatobiliary surgery providing R0 resection rate, blood loss volume and morbidity rate superimposable to referral centers. Furthermore, H&S allow a reduction of health mobility with consequent money saving for patients and institutions.

## 1 Introduction

Colorectal cancer (CRC) is the third most frequent neoplastic illness in the world, with approximately 1.4 million new cases diagnosed each year and 694.000 deaths [1,2]. The liver is the primary organ for CRC Liver metastases [3]. It is expected that the 20–25% of CRC patients shows synchronous metastases at diagnosis, and that the 60% will acquire metastases throughout follow-up [4,5]. Surgery in combination with other therapies, such as neoadjuvant or adjuvant chemoradiotherapy or the recently proposed molecular targeted therapy, is the only possibly curative option, with a considerable improvement in overall survival [6,7]. The scheduling of simultaneous hepatic and colorectal surgery has been hotly debated in recent years, with various techniques advocated by various experts [4]. Simultaneous CRC and Colo Rectal Liver Metastastases (CRLM) resections offer multiple advantages and, as established by different investigations, do not result in increased morbidity and death if compared to delayed hepatectomies with major economic and biological benefits [[8], [9], [10]]. The presence of liver metastasis has a deep impact on prognosis and management of CRC, so their early diagnosis is the aim of several multidisciplinary research groups that are investigating the application of artificial intelligence methods to early detect CRLM [6,11] also trying to predict the response to treatment. Despite laparoscopic approach showed short- and long-terms outcomes superimposable to open approach, it is still debated the role of robotic surgery in CRLM when compared to open surgery [12], especially when performed in General Units not dedicated to HPB surgery. So, our study aimed to verify the efficacy and safety of perioperative outcomes after RLR and ORL for CRLM in an Hub&Spoke Learning Program (H&S) between a high volume center for liver surgery and high volume centers for robotic surgery.

## 2 Material & methods

We retrospectively reviewed a prospectively collected database of patients undergoing liver resections at the: Hepatobiliary and Pancreatic Surgery Unit of Pineta Grande Hospital (Castel Volturno, Italy) and General and Robotic Surgery Unit of San Giovanni Battista Hospital (Foligno, Italy) and General Surgery Unit of San Donato Hospital (Arezzo, Italy) from January 2011 to December 2022. The H&S follow what was carried on in the previous years between the Robotic Surgery Unit of Spoleto and the HPB Referral Center of AORN A. Cardarelli Hospital Naples directed by Prof. L. Casciola and Prof. F. Calise [13]. Despite there was not signed any formal agreement between the two institutions, physicians discussed collegially the single cases and they moved from the two centers, with dedicated case-by-case authorizations, to improve their learning curve and to perform surgical intervention tutoring of their colleagues in the most difficult procedures. Two-hundred and sixty-eight patients underwent OLR at Pineta Grande Hospital, while 115 patients experienced a RLR at General and Robotic Surgery Unit of San Giovanni Battista Hospital and General Surgery unit of San Donato Hospital. The learning curve of Robotic Units was carried out from 2009 to 2016(13). Patients involved during robotic learning curve were excluded, in order to reduce the bias due to training period of robotic surgeons’ team. We selected, after propensity score (PS) matching, 45 consecutive liver resections for CRLM performed by both centers. Patients were divided in two cohorts: open liver resection (OLR) and robotic liver resection (RLR) groups, according to surgical approach. All patients signed an informed consent allowing the anonymous scientific use of clinical data and images. The study was carried out according to the Declaration of Helsinki guidelines and was approved by the Institutional Review Board of the University of Molise (protocol number 10/21, approved date: May 12, 2021).

### 2.1 Preoperative workup

The preoperative staging of the patients was performed with computed tomography (CT) scan of the chest and abdominal region, according to the Italian CRC guidelines [14]. In selected cases, we performed triphasic contrast liver-specific magnetic resonance imaging (MRI) to overcome CT limitations, mainly in patients who underwent neoadjuvant or adjuvant chemotherapy for CRC, considering the better detection of liver metastasis distributions and their characteristics obtained by MRI [15]. CRLM were defined as synchronous whenever diagnosed within 3 months after CRC diagnosis. An estimated liver remnant volume was performed in complex cases, in order to guarantee macroscopic negative margins, through bodyweight ratio above 0.5% [16] and future liver remnant value. In all cases, a multidisciplinary team (hepatic and gastrointestinal tumor boards) composed by oncologist, radiologists, anesthesiologists and surgeons selected patients eligible to surgical procedures. Patients affected by concomitant extrahepatic liver metastases were excluded. All cases were discussed at institutional tumor board which shared the simultaneous surgical approach.

### 2.2 Operative approach

**Fig. 1:**
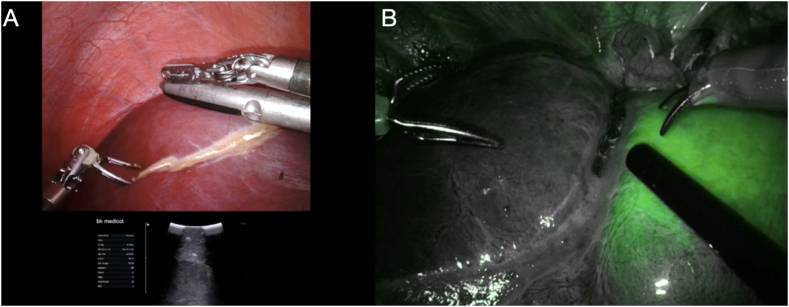
**A.** Use of Intraoperative UltraSound to identify metastases margins; **B.** Vascularization assessment during robotic liver resection after intravenous Indocyanine Green (ICG) injection.

**Fig. 2:**
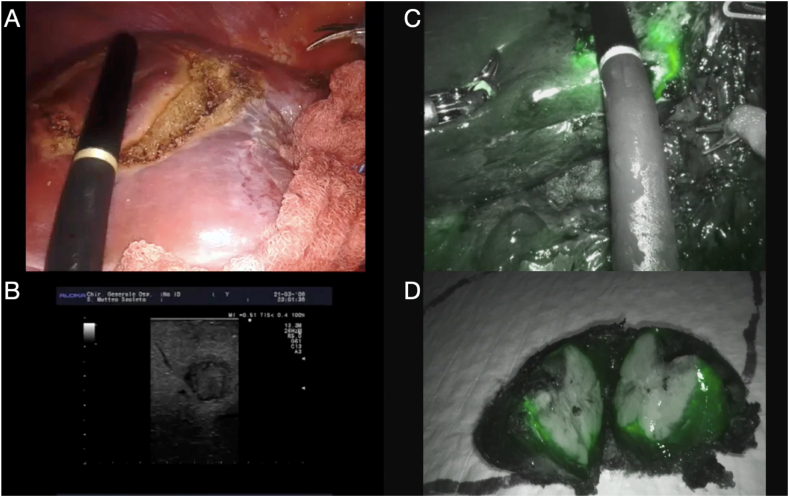
**A-B.** Intraoperative UltraSound-guided wedge resection to perform parenchymal sparing and achieve R0 margins; **C-D.** Accumulation of Indocyanine Green (ICG) around liver metastases after preoperative injection to evaluate margin status and recognize subcapsular metastases.

The Da Vinci Surgical System (Intuitive, Inc., Sunnyvale, CA), either Si and Xi system, was used under the tutoring of the experienced surgeons team [17]. Specific details on surgical approaches used for robotic liver resections have been reported [18]. Usually, during parenchymal transection, the central venous pressures and stroke volume variation were monitored and kept low, to minimize blood loss and not interfere with fluid resuscitation [19]. Furthermore, intraoperative ultrasound (IOUS) was systematically performed to confirms preoperative findings and to achieve parenchymal sparing and R0 resection [20,21]. The Cavi-Pulse Ultrasonic Surgical Aspirator (CUSA®, Model 200T, Valley Lab., Boulder, Colorado, USA) was used to obtain parenchymal transection in OLR [22,23]. In RLR group, the Aquamantis (Aquamantys®, bipolar sealer, Medtronic, Portsmouth, USA) was used to perform hepatic resection and hemostasis. In all cases, the surgeons prepared the pedicle clamping and used an intermittent Pringle Manoeuvre as depicted in Table 3, Table 4. Intrabdominal drains and recovery in the Post-Operative Intensive Care Unit (PO-ICU) postoperatively were at the discretion of the surgical and anesthesiologic teams and depicted in Table 3, Table 4. Anatomical liver resections were performed and described according to Brisbane classification [24]. We defined the major hepatectomy as the resections of ≥3 hepatic segments. Minor hepatectomies were described as excision <3 liver segments, while non-anatomic wedge resections were defined as <1 Couinaud's segment [25]. Indocyanine green (ICG) fluorescence was routinely used in RLR (Xi system) to identify single/multiple tumors , for the detection of resection margins and/or to define vascularization assessment, requiring a dose of 0.25 mg per kilogram of body weight 24 h before or during surgery following the package insert [26] *[*Fig. 1(A and B), 2(A – D)] (see Fig. 2).

### 2.3 Follow‐up

After hospital discharge, a contrast-enhanced spiral CT of the chest, abdomen and pelvis was performed every 6 months for the first 2 years and yearly thereafter. Furthermore, we evaluate serum levels of carcinoembryonic antigen (CEA) and carbohydrate antigen 19-9 (CA19.9), according to the Italian Guidelines for CRC [14].

### 2.4 Primary and Secondary Endpoints

This study primary aims to evaluate the perioperative outcomes of OLR and RLR, in order to assess the safety and effectiveness of both procedures. For this purpose, we analize the operative time (min), blood loss (ml), transfusion rate (%), use and duration (min) of the Pringle manoeuvre, ICU stay (days), length of stay (days), mortality rate (%). Therefore, we stratified postoperative complications according to Clavien-Dindo classification (CD) [27 considered them severe when ≥3. We also calculated the Comprehensive Complication Index (CCI®) [28]. We collected and defined liver-specific complications as: ascites, abdominal drainage output >10 ml per kg bodyweight per day on POD 3; biliary leakage as a bilirubin concentration 3 times greater than serum level in abdominal drainage fluid, liver failure on POD 5 [29]. Furthermore, we analyze the assessment of R0 resection (defined as resection performed at ≥1 mm from liver metastases) in patients underwent OLR and RLR [30]. Additionally, we compared outcomes of minor liver resections of ORL and RLR groups.

### 2.5 Propensity-score matching

PS matching was performed to analyze the ORL and RLR cohorts minimizing differences and bias. We obtained the PS using a logistic regression model that included: age [31,32], sex [32,33], American Society of Anesthesiologists (ASA) classification [32,34], morbidity [32], Body Mass Index (BMI) [[35], [36], [37], [38]], administration of preoperative chemotherapy according to Italian Guidelines for CRC [14,39,40], number of concomitant liver resections (single or multiple) [40], anterolateral or postero-superior segments. After estimation of PS, a regular 1:1 Nearest-Neighbour matching was performed [41].

### 2.6 Statistical analysis

Each surgeons team compiled a Microsoft Excel Database. An extensively analysis was performed to assess the data quality, then data were collected into a single database. Quantitative variables were reported as mean ± Standard Deviation (SD) or median and interquartile range (IQR). Chi-square test (χ^2^) or Fisher's exact test was performed to compare qualitative variables, while T-test was performed to analyze normal distributed variables. A two-tailed *p value* < 0.05 was recognized as statistically significant. Data analysis was carried out with IBM Statistical Package for the Social Sciences (IBM SPSS®).

## 3 Results

From January 2011 to December 2021 a total of 383 patients affected by CRLM underwent liver resection. After PS matching, we excluded 293 patients, including 45 (50 %) ORL resections and 45 (50 %) RLR. The baseline characteristics of ORL and RLR groups are summarized in Table 1. RLR was mainly performed in patients not previously undergoing abdominal surgery (*p* = 0.0001). A similar percentage of patients in the ORL and RLR had multiple liver lesions (55.56 % *vs* 48.89 % respectively). Moreover, major resections were mainly performed in patients undergoing ORL (48.49 % *vs* 22.22 %, *p* = 0.0148) (Table 2). RLR included: single non-anatomical resections (19, 42.22 %), multiple non-anatomical resections (18, 40 %), bisegmentectomy (3, 6.67 %), single anatomical resections (2, 4.45 %), right hepatectomy (1, 2.22 %), left hepatectomy (1, 2.22 %), left lobectomy (1, 2.22 %). ORL were: right trisectionectomies (6, 13.33 %), right hepatectomy (3, 6.67 %), left hepatectomy (5, 11.11 %) single non-anatomical resections (13, 28.89 %), multiple non-anatomical resections (13, 28.89 %), right lobectomy (4, 8.89 %), explorative laparotomy (1, 2.22 %). Four (4/45, 8.89 %) robotic procedures were converted as open resections: in one case (25 %) due to diaphragmatic infiltration, in one case (25 %) due to hepatic pedicle infiltration, in one case (25 %) due to adherence, in one (25 %) case due to the huge number of liver metastases.

**Table 1:** Baseline characteristics of patients underwent CRLM resection in open and robotic centers. OLR, open liver resection; RLR, robotic liver resection; BMI, Body Mass Index; ASA, American Society of Anesthesiologists; COPD, Chronic Obstructive Pulmunary Disease.

	After matching			
	All	OLR	RLR	*p value*
Number of patients, (%)	90 (100)	45 (50)	45 (50)	*NA*
Age; years; mean ± SD	66.99 ± 10.68	67.44 ± 8.35	66.53 ± 12.67	*0.6884*
Female gender; n. (%)	37 (41.11)	17 (37.78)	20 (44.44)	*0.6686*
BMI; kg/m^2^; mean ± SD	26.11 ± 3.84	26.27 ± 3.89	25.95 ± 3.83	*0.6951*
ASA score; median (IQR)	2 (1-4)	2 (1-4)	2 (1-4)	*NA*
Cardiac disease; n. (%)	12 (26.67)	9 (20)	3 (6.67)	*0.1184*
COPD; n. (%)	2 (13.33)	1 (2.22)	1 (2.22)	*NA*
Hypertension; n. (%)	40 (44.44)	24 (53.33)	16 (35.56)	*0.1371*
Diabetes mellitus; n. (%)	10 (11.11)	9 (20)	1 (2.22)	***0.0151***
Previous abdominal surgery; n. (%)	46 (51.11)	33 (73.33)	13 (28.89)	***0.0001***
Preoperative chemotherapy; n. (%)	8 (8.89)	6 (13.33)	2 (4.45)	*0.2663*

**Table 2:** Variables of patients underwent CRLM in OLR and RLR. OLR, open liver resection; RLR, robotic liver resection.

	After matching			
	All	OLR	RLR	*p value*
Number of patients, (%)	90 (100)	45 (50)	45 (50)	*NA*
Extent of liver resection - Minor, n. (%) -Major, n. (%)	58 (64.44) 32 (35.56)	23 (51.11) 22 (48.49)	35 (77.78) 10 (22.22)	***0.0148***
Number of liver lesions -Solitary, n (%) -Multiple, n. (%)	43 (47.78) 47 (52.22)	20 (44.44) 25 (55.56)	23 (51.11) 22 (48.89)	*0.6732*
Size of biggest resection -<5 cm, n. (%) -≥5 cm, n. (%)	77 (85.56) 13 (14.44)	34 (75.56) 11 (24.44)	43 (95.56) 2 (4.44)	***0.0139***
Tumor location according to segments -Anterolateral, n. (%) -Posterosuperior, n. (%)	42 (46.67) 48 (53.33)	22 (48.89) 23 (51.11)	20 (44.44) 25 (55.56)	*0.8328*
Simultaneous resection of colorectal cancer and liver metastases, n. (%)	31 (34.44)	9 (20)	22 (48.89)	***0.0073***
Location of primary -Right colon, n. (%) -Left colon, n. (%) -Rectum, n. (%)	24 (26.67) 32 (35.56) 24 (26.67)	13 (28.89) 17 (37.78) 15 (33.33)	11 (24.45) 15 (33.33) 19 (42.22)	*0.8120* *0.8259* *0.5146*
Type of metastases -Synchronous, n. (%) -Metachronous, n. (%)	34 (37.78) 56 (62.22)	14 (31.11) 31 (68.89)	20 (44.44) 25 (55.56)	*0.2769*
Type of disease -Unilobar, n. (%) -Bilobar, n. (%)	59 (66.56) 31 (34.44)	24 (53.33) 21 (46.67)	35 (77.78) 10 (22.22)	***0.0258***

**Table 3:** Intra and postoperative outcomes of patients underwent liver resections in ORL and RLR. OLR, open liver resection; RLR, robotic liver resection; PO-ICU, Post-Operative Intensive Care Unit; PHLF, Post-Hepatectomy Liver Failure.

	After matching			
	All	OLR	RLR	*p value*
Number of patients, (%)	90 (100)	45 (50)	45 (50)	*NA*
Operative time; mean, min ±SD	312.13 ± 109.47	322.15 ± 131.94	302.11 ± 102.99	*0.4240*
Blood loss, ml	275.11 ± 38.74	321.56 ± 48.12	226.67 ± 29.36	***0.0001***
Pringle maneuverer -Yes, n. (%) -Mean (min)	40 (44.44) 38.06	20 (44.45) 45	20 (44.45) 31.12	*1.0000*
Conversion rate, n. (%)	4 (4.44)	–	4 (8.89)	–
Transfusion rate, n. (%)	5 (5.56)	5 (11.11)	0 (0)	*0.0556*
PO-ICU stay, n. (%)	26 (28.89)	13 (28.89)	3 (6.67)	***0.0113***
Hospital stay; mean days (IQR)	11.90 (3–104)	15 (4–104)	8.8 (3-34)	***0.0001***
PHLF, n. (%) -No -Yes	89 (98.89) 1 (1.11)	44 (97.78) 1 (2.22)	45 (100) (0)	*1.0000*
Bile leak -No -Yes	90 (100) 0 (0)	0 (0) 0 (0)	0 (0) 0 (0)	*1.0000*
Clavien-Dindo Classification -I-II, n. (%) - ≥ III, n. (%)	76 (84.44) 14 (15.56)	37 (82.22) 8 (17.78)	39 (86.67) 6 (13.33)	*0.7722*
Comprehensive Complication Index, mean (range)	16.3 (8.7–26.2)	16.7 (8.7–26.2)	15.9 (8.7–26.2)	*0.5939*
30-day mortality rate, n. (%)	3 (3.33)	3 (6.67)	0 (0)	*0.2416*
R0 resection, n. (%)	85 (97.78)	45 (100)	43 (95.55)	*0.4944*

**Table 4:** Baseline characteristics, intra and postoperative outcomes of patients underwent minor liver resections in ORL and RLR. OLR, open liver resection; RLR, robotic liver resection; BMI, Body Mass Index; ASA, American Society of Anesthesiologists; PO-ICU, Post-Operative Intensive Care Unit; PHLF, Post-Hepatectomy Liver Failure.

	OLR	RLR	*p value*
Number of minor liver resection, (%)	23/45 (51.11)	35/45 (77.78)	***0.0148***
Age; years; mean ± SD	66.13 ± 9.85	67.16 ± 11.98	*0.7330*
Female gender; n. (%)	16 (69.56)	14 (40)	***0.0345***
BMI; kg/m^2^; mean ± SD	28.11 ± 7.91	26.05 ± 4.07	*0.2276*
ASA score; median (IQR)	2 (1-4)	2 (1-4)	*NA*
Operative time; mean, min ±SD	261.13 ± 97.67	297.20 ± 100.85	*0.1820*
Blood loss, ml	303.44 ± 76.83	245.38 ± 27.62	***0.0001***
Pringle maneuverer -Yes, n. (%) -Mean (min)	13 (56.52) 30	17 (48.57) 27.86	*0.6001*
Conversion rate, n. (%)	–	4 (11.43)	–
Transfusion rate, n. (%)	1 (4.35)	0 (0)	*0.3966*
PO-ICU stay, n. (%)	6 (26.10)	3 (8.57)	*0.1450*
Hospital stay; mean days (IQR)	11 (4-26)	8.8 (3-34)	*0.5555*
PHLF, n. (%) -No	0 (0)	0 (0)	*1.0000*
Bile leak -No	0 (0)	0 (0)	*1.0000*
Clavien-Dindo Classification -I-II, n. (%) - ≥ III, n. (%)	22 (95.65) 1 (4.35)	32 (91.43) 3 (8.57)	*1.000*
Comprehensive Complication Index, mean (range)	15.8 (8.7–26.2)	14 (8.7–26.2)	*0.2867*
30-day mortality rate, n. (%)	0 (0)	0 (0)	*1.000*
R0 resection, n. (%)	23 (100)	33 (94.28)	*0.1443*

### 3.1 Perioperative outcomes

Matching cohort analysis PSM showed shorter operative time in patients underwent RLR without stastical differences (*p* = 0.4240). Although, a less blood loss rate (*p <* 0.001) was reported in the RLR group, Pringle maneuver was adopted in the 44.45 % (20 patients) of resections in both surgical approaches with similar duration. According to these findings, red blood cells transfusion was necessary for only ORL (11.11 % *vs.* 0 %, *p =* 0.0556). Perioperative outcomes are listed in Table 2, Table 3 Postoperative ICU stay was required for only 3 patients undergone RLR (6.67 % *vs*. 28.89 % respectively RLR and OLR) (*p* = 0.0113). Concerning the hospital stay we found 15 days LOS for OLR vs 8.8 days for RLR group (*p* = 0.001). 30-day mortality rate was higher in ORL cohorts (3 *vs.* 0 patients); 1 (1/45, 2.22 %) patient died due to liver failure after right hepatectomy, 2 (2/45, 4.44 %) patients died due to non-surgical complications. The open surgery team achieved in all cases (100 95.55% of RLR achieved R0 resection. The not R0 resections were 4.45% of R1vasc margins. 11.11% of RLR achieved R1vasc margins before PS.

## 4 Discussion

PS showed that RLR for CRLM, especially within Hub and spoke learning program (H&S), is safe and effective providing perioperative outcomes comparable to OLR. Our findings show how collaborative effort offers better results in the management of CRLM allowing the best care for patients besides an iterative learning curve for physicians belonging to HPB and Robotic General Surgery Units. To our knowledge, our cohort study is one of the largest series of patients undergoing OLR and RLR for CRLM after applying PSM method in H&S learning program. The 2 cohorts are homogeneous either for baseline characteristics either for surgical complexity.

### 4.1 Hub&Spoke training programme

Surgical education has traditionally transferred from experienced surgeons (high volume centers) to trainees (low volume centers and young surgeons) [[42], [43], [44]]. However, the H&S offers a paradigm change in surgical education: the Hub offers mentorship, ensuring standardization of training, while spoke unites benefit of huge cohort of patients and hands-on clinical experience [13,45,46]. Consequently, decentralization and collaboration programs between H&S Unites achieve an adequate experience in spoke surgeons also to perform major surgical procedures as HPB surgery [13,47]. Conserning patients’ point of view, the H&S increases access to surgical education in underserved or remote areas, allowing trainees to learn and perform surgical procedures in their local Units. This approach is very useful for the managing of CRLM because, often primary cancer is diagnosed and treated in peripheral centers, so the chance to treat CRC simultaneously to LM allow to reduce health mobility to HPB referral centers with the sparing of related costs [46,47].

Our study model is based on H&S: the HPB surgeon tutor (Castel Volturno team) has overseen pre-, intra-e post-operative course of patients to train robotic surgeon team (Foligno team) and the other way around. Multidisciplinary online conferences were conducted to discuss patients’ data. However, in all cases the discussion included an extensively evaluation of patient fitness to surgery and a lengthily study of radiological imaging. Depending upon the complexity of surgical resection, HPB surgeon assisted during the RLR, having also the chance to improve his ability in minimally invasive approaches. The postoperative management of patients was carried out by sharing clinical data, blood tests and radiological data day-by-day to treat any complications adequately. The H&S involved either physicians either nurses of both centers. After H&S experience, both teams were considered trained in open and robotic liver surgery after performing around 50 LR as highly reported in literature [[48], [49], [50]]. We have to underline that after the H&S learning program the two equips deeply improved their surgical skills allowing a reduction of tutor needing for the minor complex procedures. Furthermore, during learning curve period the mentor actively performed one-on-one liver resections, while the second part of the program involved the presence of the mentor in the operating room.

### 4.2 Open vs. minimally invasive surgery

Contrary to previous laparoscopic surgery experiences, OLR for CRLM is preferred in the literature in major hepatectomies compared to RLR [49,[51], [52], [53]]. This trend is also verified in our cohort selecting the most challenging procedures for the open approach as already reported [54]. Nevertheless, it should be underlining that similar findings were observed in terms of number of liver lesions (solitary or multiple, p = 0.6732) and tumor locations (anterolateral or posterosuperior segments, p = 0.8328). These data point out that open surgery was widely performed in more than ≥5 cm and ≥3 segments resections. Given the dissimilar distribution of major and minor hepatectomies in the two matched groups, and the similar proportion of lesions located in posterosuperior segments, it seems reasonable to conclude that these are accurate intraoperative outcomes if the robotic approach is applied in complex liver resections, but not considering major or minor resection score. To overcome this bias, we analyzed the outcomes of minor resection in ORL and RLR groups (Table 4). As depicted in Table 4 peri-operative outcomes of RLR and ORL are superimposable, except for blood loss which benefits of the minimally invasive approach. In literature have been demonstrated the benefits of RLR regarding less intraoperative blood loss, lower morbidity rate, faster discharge and better functional activity after surgery compared to OLR [17,18,[55], [56], [57], [58], [59]]. Considering the impact of frailty in HPB patients, no age differences were founded between the groups [60,61]. After PSM, patients in RLR experienced lower blood loss, ICU admission and length of hospital stay (p = 0.0001), while similar operative time were collected (322.15 ± 131.94 vs. 302.11 ± 102.99 in ORL and RLR respectively). Our results are in line with others experiences [17]. From the point of view of the health system, these results have achieved that higher technology expenses are beneficial reducing ICU and LOS with a benefit on the costs impact of the National Health System. Post hepatectomy liver failure (PHLF) was reported in 1 (2 %) case of OLR, may be due to fatty infiltration of the liver after irinotecan-based chemotherapy [62], in patient undergone a huge hepatic resection, despite the pre-operative work-up allowed a sufficient function of the liver remnant. As depicted in the Results section, there was a striking positive oncologic effect of the minimally invasive approach, particularly in patients with unilobar or smaller than 5 cm diameter CRLM. Nevertheless, The R0 resection rate was 100 % for ORL and 95.55% for RLR group.Similar findings are reported in literature [63]. We could not investigate the real costs due to the deviceprice which is variable in the huge period analyzed (from 2011 to 2022) and in two different region of Italy. As largely reported in literature, robotic surgery is more expensive compared to open and laparoscopic surgery. Higher purchase and maintenance costs, higher price of consumable surgical supplies contribute to increase robotic costs. Many authors reported an increasing up to 10 % of surgery expense when compared to laparoscopic surgery [64,65]. In our experience, the RLRs were performed without CUSA® and, in only very few cases, using mechanical stapler or other expensive devices. In our cohort of patients, >90 % of RLR was performed trough 3 surgical devices that included bipolar and scissors. Furthermore, our experience shows that well-trained surgeons and experiences in liver surgery are related to costs. It contributedhas been contributed to reduce the final costs of surgery when added to cost of lower length of stay after minor and major liver surgery. Stewart et al. [66] compared data of 87 patients who underwent minor liver resections (robotic n = 46, open n = 41). The authors concluded that specimen size, operative time, estimated blood loss, and margin status were similar for RLR and OLR groups. Nevertheless, lower complication rate and length of stay were reported for RLR group (p < 0.001). After a multivariate analysis, the authors reported a lower cost for minor RLR costing $534 less than open resections may be due to superior peri-operative outcomes. Similar findings are reported by Daskalaki et al. [64] who analyzed data about 68 patients undergoing RLR and 55 ORL. They concluded that RLR improved overall morbidity and ICU rates and reduced the hospital stay. As result, robotic procedures are financially comparable to ORL. Knitter et al. [67] analyzed costs and perioperative outcomes of 146 patients who underwent open, laparoscopic and robotic major liver resection. The estimated median price for one-day of recovery was 1725 € in the robotic group, 1633 € in the laparoscopic group and 1205 € in the open group, (*p* < 0.0001). Furthermore, median daily and total costs were superimposable for robotic and laparoscopic approach (16,648 € vs. 14,578 €). Considering these findings, the authors have been settled that RLR represents a valid alternative to laparoscopic surgery also in case of major liver resections as reported in other surgical fields [18,68,69]. We suggest additional prospective studies on this issue. The H&S showed a highly satisfaction rate of patients, the surgical collaboration reduced costs due to health migration especially in patients who experienced prolonged LOS and improved the earlier discharge thanks to a cropped distance from the reference care hospital. Regarding laparoscopic liver surgery, we can conclude that short- and long-terms outcomes are superimposable when compared to RLR [59,[70], [71], [72], [73]]. Tsilimigras et al. [74] reviewed 31 studies in order to analyze the safety and oncologic outcomes of RLR. They include more than 1100 patients and conclude that robotic technique for liver resections is a safe alternative to laparoscopic surgery either for minor either for major resections in terms of blood loss, operative time, hospital stay and R0 rates. Nevertheless, authors report a lower complication rate in laparoscopic liver resection (17.6 % and 5.9 % in RLR and laparoscopic group respectively). Similar findings were reported by Tsung et al. [71], Aboudou et al. [75] and Kamarajah et al. [76]. Analyzing RLR outcomes in primary and metastatic liver tumors, in literature is reported a better perioperative tolerability when compared to OLR. Di Benedetto et al. [77] performed a PSM comparing patients with HCC underwent RLR and ORL on the basis of baseline characteristics, surgical difficulty and preoperative risk of tumor recurrence. Patients underwent RLR experienced shorter hospital lenght of stay and PHFL rate and lower complications rate. Nevertheless, OLR group showed shorter operative times and lower blood loss. Other literature experiences reported similar data [78]. Chang et al. [79] reported data of 171 patients enrolled in a Randomized Clinical Trial. 86 experienced RLR and 85 ORL for simultaneous rectal cancer and liver metastases. The authors described fewer complications within 30 days after surgery in RLR when compared to ORL (31.4 vs. 57.6 %, respectively). RLR group experienced lower blood loss, faster bowel function recovery and shorter hospital stay. In terms of survival, the 3-year disease-free survival rate (39% vs. 35%) and the 3-year overall survival rate (76 % vs. 72 %) were not statistically significant between the two groups.

## 5 Limitations

Although the partnership obtained for open and robotic surgical team showed encouraging postoperative outcomes, it is important underline that the health system did not define specific rules to continue this program despite the effort of the surgeons involved.

## 6 Conclusion

PSM showed that RLR for CRLM especially in an H&S learning program is safe and effective providing a postoperative complication rate comparable to OLR, with a lower blood loss and morbidity rates, and shorter postoperative hospital stay in selected patients. Furthermore, the H&S allowed a reduction of health mobility with consequent money saving for patients and for the Health Care System. The H&S has the potential to improve surgical education and collaboration between centers. Finally, the technology advances, including telemedicine and virtual reality platforms, can further enhance the educational experience of surgeons.

## Funding

The authors received no financial support for the research, authorship, and/or publication of this article.

## Institutional Review Board statement

The authors are accountable for all aspects of the work in ensuring that questions related to the accuracy or integrity of any part of the work are appropriately investigated and resolved. Informed consent: Informed consent has been obtained from all individuals included in this study. The study was conducted according to the guidelines of the Declaration of Helsinki and approved by the Institutional Review Board of University of Molise (protocol number 10/21, approved date: May 12, 2021).

## Informed consent statement

Informed consent was obtained from all subjects involved in the study.

## Data availability statement

The datasets used and/or analyzed during the current study are available from the corresponding author on reasonable request.

## Additional information

No additional information is available for this paper.

## CRediT authorship contribution statement

**Aldo Rocca:** Writing – review & editing, Visualization, Validation, Supervision, Resources, Project administration, Methodology, Investigation, Formal analysis, Data curation, Conceptualization. **Pasquale Avella:** Writing – original draft, Visualization, Validation, Supervision, Software, Resources, Project administration, Methodology, Investigation, Formal analysis, Data curation, Conceptualization. **Andrea Scacchi:** Methodology, Investigation, Data curation. **Maria Chiara Brunese:** Investigation. **Micaela Cappuccio:** Methodology, Investigation, Data curation. **Michele De Rosa:** Investigation. **Alberto Bartoli:** Investigation. **Germano Guerra:** Supervision. **Fulvio Calise:** Supervision. **Graziano Ceccarelli:** Writing – review & editing, Writing – original draft, Validation.

## Declaration of competing interest

The authors declare that they have no known competing financial interests or personal relationships that could have appeared to influence the work reported in this paper.
